# Closure of Wounds

**Published:** 1903-11

**Authors:** 


### Closure of Wounds.

Porter (*Journal of the American Medical Association,* July 25th) emphasizes the following points:

1. The use of sutures should be avoided save where necessity demands their use. Many wounds, in which sutures are now commonly used, may be coapted more perfectly and more safely without the use of sutures.

2. Tension and moisture are the only conditions making sutures necessary.

3. When sutures are necessary, buried absorbable suture should be used in all cases where there is no infection.

4. The necessity for drainage does not contraindicate the use of adhesive plaster for purposes of coaptation.

5. It is doubtful if non-absorbable suture material should ever be used with a view to its remaining permanently.

6. Non-absorbable sutures are not necessary nor advisable save in intestinal work and in the presence of sepsis.

7. In those cases in which non-absorbable sutures are necessary that method of applying them should be chosen which will subject the tissues for infection through the skin, and permit of their being removed when they have fulfilled their mission.

---

Scene: Office of a pompous doctor who knows it all. Enter, a tired man, who drops in a seat and says that he wants treatment. The doctor puts on his eye-glasses, looks at his tongue, feels of his pulse, sounds his chest, and then draws up to his full height and says: "Same old story, my friend. Men can't live without fresh air. No use trying it. I could make myself a corpse, like you are doing by degrees, if I sat down in my office and didn't stir. You must have fresh air; you must take long walks, and brace up by staying out doors. Now, I could make a drug store of you, and you would think I was a smart man, but my advice to you is to walk, walk, walk."

Patient—"But, doctor——"

Doctor—"That's right. Argue the question. That's my reward. Of course you know all about my business. Now, will you take my advice? Take long walks every day, several times a day, and get your blood in circulation."

Patient—"I do walk, doctor. I——"

Doctor—"Of course you walk. I know that; but walk more. Walk ten times as much as you do. That will cure you."

Patient—"But my business——"

Doctor—"Of course, your business prevents it. Change your business, so that you will have to walk more. What is your business?"

Patient—"I am a letter-carrier."

Doctor (paralyzed)—"My friend, permit me once more to examine your tongue."—*Hot Stuff.*

---

A Nebraska editor stated that a certain girl's breast was filled with rage, and that wise guy, the printer, got it "rags," and now the editor is camping out on a rise northwest of the town, where he can get a good view of the landscape from four different directions.—*Selected.*

